# Dorso-Lateral Frontal Cortex of the Ferret Encodes Perceptual Difficulty during Visual Discrimination

**DOI:** 10.1038/srep23568

**Published:** 2016-03-30

**Authors:** Zhe Charles Zhou, Chunxiu Yu, Kristin K. Sellers, Flavio Fröhlich

**Affiliations:** 1 Department of Psychiatry, University of North Carolina at Chapel Hill, 27599, Chapel Hill NC, USA; 2 Neurobiology Curriculum, University of North Carolina at Chapel Hill, 27599, Chapel Hill NC, USA; 3 Department of Cell Biology and Physiology, University of North Carolina at Chapel Hill, 27599, Chapel Hill NC, USA; 4 Department of Biomedical Engineering, University of North Carolina at Chapel Hill, 27599, Chapel Hill NC, USA; 5 Neuroscience Center, University of North Carolina at Chapel Hill, 27599, Chapel Hill NC, USA; 6 Department of Neurology, University of North Carolina at Chapel Hill, 27599, Chapel Hill NC, USA.

## Abstract

Visual discrimination requires sensory processing followed by a perceptual decision. Despite a growing understanding of visual areas in this behavior, it is unclear what role top-down signals from prefrontal cortex play, in particular as a function of perceptual difficulty. To address this gap, we investigated how neurons in dorso-lateral frontal cortex (dl-FC) of freely-moving ferrets encode task variables in a two-alternative forced choice visual discrimination task with high- and low-contrast visual input. About two-thirds of all recorded neurons in dl-FC were modulated by at least one of the two task variables, task difficulty and target location. More neurons in dl-FC preferred the hard trials; no such preference bias was found for target location. In individual neurons, this preference for specific task types was limited to brief epochs. Finally, optogenetic stimulation confirmed the functional role of the activity in dl-FC before target touch; suppression of activity in pyramidal neurons with the ArchT silencing opsin resulted in a decrease in reaction time to touch the target but not to retrieve reward. In conclusion, dl-FC activity is differentially recruited for high perceptual difficulty in the freely-moving ferret and the resulting signal may provide top-down behavioral inhibition.

Prefrontal cortex (PFC) is widely interconnected with other cortical and sub-cortical areas^1^,^2^,^3^,^4^. Neurons in PFC are therefore ideally situated to (1) prioritize and integrate sensory signals, (2) prepare behavioral responses, and (3) assess outcomes. Indeed, PFC neurons encode a broad range of task-related signals. PFC neurons preferentially respond to behaviorally relevant sensory input^5^,^6^,^7^, provide top-down control signals that shape sensory processing by allocating attention in preparation for goal-directed actions^8^,^9^,^10^,^11^, and provide behavioral inhibition to overcome habitual responses^10^,^12^,^13^,^14^,^15^.

Together, these findings support a model in which goal-directed behavior arises through the interaction of bottom-up signals from sensory areas and top-down control signals from PFC. Sensory discrimination tasks provide a behavioral assay of how bottom-up sensory signals are processed and how they lead to goal-directed behavior. Indeed, visual discrimination between two stimuli is a common element of many tasks that are used to study the neuronal correlates of perception. Accordingly, the role of (cortical) visual areas in tasks that require discrimination between different visual stimuli has been extensively studied^16^,^17^,^18^,^19^. In contrast, the role of PFC in visual discrimination, in particular as a function of perceptual difficulty, has remained mostly unstudied. To address this gap, we investigated the neuronal spiking dynamics in dorso-lateral frontal cortex (dl-FC) of freely-moving ferrets during a two-alternative forced choice, visual discrimination task with two levels of perceptual difficulty. We hypothesized that dl-FC neurons dynamically encode task variables and that they are differentially recruited in trials with high perceptual difficulty. We further hypothesized that this brain area is causally involved in performing the discrimination task.

To test these hypotheses, we trained ferrets to perform a touchscreen-based visual discrimination task. In this task, the animals initiated trials, selected the conditioned stimulus out of two simultaneously presented images, and retrieved a water reward at the end of successful trials. One group of animals was implanted with electrode arrays in left dl-FC^20^ for recording single-unit action potentials. We first investigated the preference of neuronal spiking responses by fitting linear models that predicted binned (instantaneous) firing rate as a function of the task difficulty (“easy” or “hard” corresponding to “high” and “low” visual contrast) and the target location on the touchscreen (“left” or “right”). We next used support vector machine analysis to assess how well the population activity encoded these task properties^21^,^22^. In a second group of animals, we expressed ArchT^23^,^24^ in dl-FC to elucidate the functional implications of dl-FC activity during this task.

## Material and Methods

All animal procedures were performed in compliance with the National Institutes of Health guide for the care and use of laboratory animals (NIH Publications No. 8023, revised 1978) and approved by the Institutional Animal Care and Use Committee of the University of North Carolina at Chapel Hill.

### Behavioral Task

**Figure 1:**
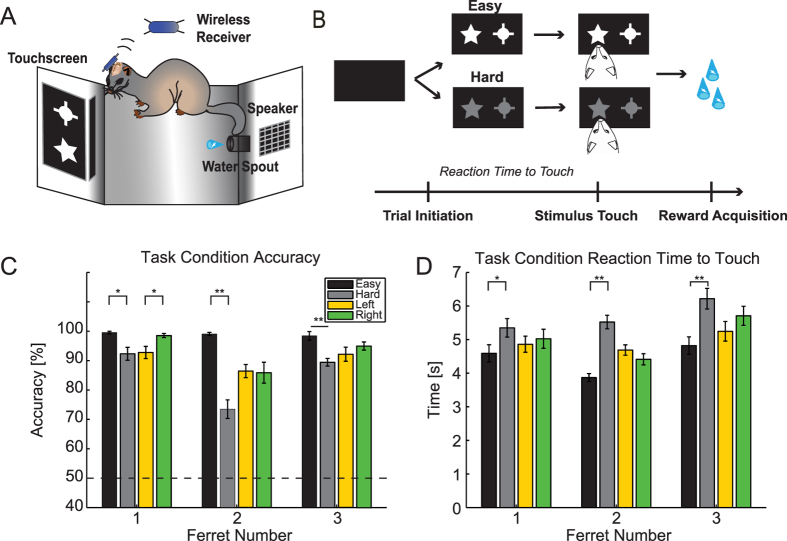
Experimental task design and behavioral performance. (**A**) Operant behavioral chamber. Ferrets were trained to perform a visual discrimination task in an operant chamber fitted with a touchscreen monitor. Ferrets initiated trials by nose-poking an infrared sensor positioned within the water spout in the rear of the chamber. Upon water release after nose-poke of the correct stimulus, the animal returned to the spout for reward acquisition. Neural activity was recorded using a wireless headstage. Visual stimuli shown in the figure are similar to the ones used in the study. (**B**) Two-alternative forced choice, visual discrimination task. Upon initiation of a trial at the water spout, an image pair was simultaneously presented in the left and right windows of the touchscreen. Trials were randomly interleaved with easy and hard image pairs. Nose-poke to the conditioned stimulus window triggered a tone and a water reward at the spout. (**C**) Mean accuracy performance in “easy” (black), “hard” (gray), “left” (yellow), and “right” (green) trials for each ferret. The dashed line represents chance performance. Error bars, standard error of the mean (SEM) across sessions. *p < 0.05; **p < 0.01. (**D**) Mean reaction time to target touch in easy (black), hard (gray), left (yellow), and right (green) trials for each ferret. Error bars, SEM across trials. *p < 0.05; **p < 0.001.

Spayed adult female ferrets (*Mustela putoris furo*, n = 3 for electrophysiology experiments, n = 5 for optogenetics experiments; group housed in a 12 hr light/12 hr dark cycle) were trained to perform a two-choice visual discrimination task. The task was carried out in a custom-made sound-attenuated behavioral box fitted with a touchscreen monitor (IRTOUCH, Beijing, China) to display stimulus images and to record nose-poke responses (Fig. 1A). A black Plexiglas sheet with left and right square cutouts was mounted in front of the touchscreen. Auditory tones were delivered through a speaker (HP Compact 2.0 Speaker) mounted on the opposite wall. A spout for water delivery and an infrared sensor to detect nose-poke initiation were positioned in the same wall 5 cm above the floor. A houselight mounted on the ceiling was turned on for the duration of the session, except during incorrect trials.

The task was adapted from an established behavioral protocol, and consisted of five training stages^25^,^26^. Each ferret learned to nose-poke one image (conditioned stimulus, CS+) of a pair to obtain water. The ferrets initiated each trial by nose-poke of the infrared sensor (Fig. 1). Following initiation, each image within the pair was simultaneously presented in the left or right windows of the touchscreen monitor (geometric black and white images from the Microsoft Clipart Gallery). CS+ and CS− locations were randomized for each trial with the added contingency that the CS+ could not appear in the same location for more than three consecutive trials. Upon nose-poke of the CS+ window, the stimuli were extinguished, an auditory pure tone (200 Hz, 63.5 dB, 500 ms duration) was played, and a water reward was released at the back of the behavioral chamber. The reaction time to touch the stimulus (time from trial initiation to stimulus touch), and thus the duration of stimulus presentation, depended on when the animal nose-poked the screen. Incorrect responses resulted in extinguishing the houselight for five seconds. For electrophysiological experiments, trials with high and low contrast versions of the image pair were randomly interleaved to assess behavior and network dynamics for low (“hard”) and high (“easy”) signal-to-noise sensory inputs. In a typical session, the animal would complete 56 trials consisting of equal numbers of all four trial types (“easy, CS+ left,” “easy, CS+ right,” “hard, CS+ left,” and “hard, CS+ right” trials). For the first session, image contrasts in the hard condition were set based on an initial calibration session where accuracy was assessed for different contrast levels. For each animal, the image contrast that produced around 75% accuracy was designated for the first test session. For subsequent sessions, image contrast for the hard condition was adjusted based on the performance in the previous session such that the accuracy of each ferret was within the range of 60% to 90%. The goal of this calibration was to make sure that the hard trials remained difficult enough such that the animal would make mistakes throughout the session.

Prior to behavioral training and testing, the animals were water restricted to enhance motivation to perform the task (5 days on water restriction/2 days off). Water sources were removed from animal home cages each Sunday before weekdays of behavioral testing. The water intake of each animal was maintained at 60 mL/kg/day as a sum of the water received during the behavioral task and supplemental water at the end of the day. Water sources were returned to the animal home cages on Friday night. Ferrets were trained twice a day (50 trials per session) and performed the final task once a day in the afternoon.

### Surgery and Electrode Implantation

Electrode implantation surgery was performed for each animal (n = 3) after completion of behavioral training. After initial anesthesia induction with intramuscular (IM) injection of ketamine/xylazine (30 mg/kg of ketamine, 1–2 mg/kg of xylazine), ferrets were intubated and deep anesthesia was maintained with isoflurane (0.5–2% in 100% oxygen). Throughout the procedure, partial oxygen saturation, end-tidal CO_2_, electrocardiogram, and rectal temperature were monitored. The body temperature was maintained at 38–39 °C and end-title CO_2_ at 30 to 50 mmHg. Using aseptic technique, tissue and muscle were resected to expose the skull surface. A small craniotomy was made above the anterior sigmoid gyrus (5 mm anterior of bregma and 2 mm lateral to the midline). Sixteen channel micro-electrode arrays (tungsten electrodes oriented in a 2 by 8 fashion, Innovative Neurophysiology, Durham, NC) were positioned above the craniotomy using a stereotaxic arm, gradually lowered to target deep layers of cortex, and fixed with dental cement. Muscle and tissue around the implant were then sutured together. Following surgery, animals were allowed to recover in their home cage for two weeks before behavioral testing. Animals were administered meloxicam for pain relief (0.2 mg/kg IM injection) and antibiotics (enrofloxacin, 5 mg/kg IM injection) during recovery.

### Viral Delivery and Fiber Implantation

The viral delivery and optical fiber implantation surgery was performed in a separate set of animals (n = 5) once the behavioral training (identical to the one for animals for electrophysiology) was complete. Similar asceptic surgery procedures as for the electrode implantation were used. For the virus delivery, we prepared either rAAV5-CamKII-ArchT-GFP (titer of 7.5 × 10^12^ vg/ml; UNC Vector Core, Chapel Hill, NC) or rAAV5-CamKII-GFP (titer of 6 × 10^12^ vg/ml; UNC Vector Core, Chapel Hill, NC) constructs in a 1 μL Hamilton syringe (Hamilton Company, Reno, NV) prior to injection. At the location of electrode implantation in the first set of animals, 1 μL of virus was delivered (0.1 μL/min) bilaterally at a depth of 0.9 mm below the surface of cortex. Ferrules with 200 um fibers (validated with >80% transmission) were positioned above the virus delivery location and secured using dental cement. A custom designed plastic cylinder implant was cemented around the ferrule to provide stability during behavior. Custom fiber implants and patch cables were fabricated according to previously published guidelines^27^.

### Optogenetic Experiments

Animals that had undergone virus injection and fiber implantation (ArchT: n = 3, GFP: n = 2, one ArchT animal was excluded from the analysis due to only minimal virus expression determined by post-mortem histology) were subject to optogenetic experiments four weeks post-surgery. Animals were water-restricted and essentially performed the same visual discrimination task as the animals implanted with recording electrodes. The task structure differed in that each session was composed of (1) counterbalanced trials with stimulation or no stimulation and, (2) a single difficulty level (high or low contrast). A 532 nm laser (GL532T3-150; Shanghai Laser & Optics Century, Shanghai, China) coupled to an optical commutator (Doric, Quebec, Canada) was used to deliver green light to the neuron population of interest. Custom-made or commercially available (Doric, Quebec, Canada) patch cables from a 1 × 2 rotary commutator (Doric, Quebec, Canada) were secured to the ferrule implant with a ceramic sleeve and stabilized with the plastic cylinder implant. Prior to each behavioral session, optical emission from the patch cable fiber tips was calibrated to 15–25 mW. For stimulation trials, the laser was turned on for a time window that ranged from trial initiation to stimulus touch (constant stimulation over the duration of stimulus presentation, also referred to as reaction time to touch). For a subset of the sessions, stimulation was applied from stimulus touch to reward acquisition. Prior to behavioral data analysis, trials where reaction time to touch exceeded 15 seconds were excluded. For the reaction time to touch analysis, we exclusively analyzed trials with correct responses. For each animal, we analyzed mean across-session accuracy, and across-trial reaction time to touch or drink for each condition (grouped by stimulation/no-stimulation and difficulty). Two-way ANOVA tests were performed on accuracy (across- session) and reaction time (pooled across trials and sessions) data. Significant results were determined based on a threshold alpha level of 0.05. Significance shown in figures was determined post-hoc using Tukey’s honest significant difference test.

### Histology

When animals reached their scientific end-point, electrolytic lesions were produced by passing current (5 μA, 10 s, unipolar) through the middle and outer metal electrodes of the recording array. Animals were then humanely euthanized with an overdose of sodium pentobarbital and immediately perfused with 0.1 M PBS and 4% paraformaldehyde solution in 0.1 M PBS. For histological verification of electrode recording sites, brains were segmented into 60 micron slices using a cryostat (CM3050S, Leica Microsystems). Brain slices were then washed with 0.1 M PBS and stained for cytochrome oxidase^28^,^29^. Slides were subsequently imaged using a widefield microscope (Nikon Eclipse 80i; Nikon Instruments, Melville, NY).

Tissue from animals used in the optogenetic experiment was segmented into 50 micron slices and mounted with DAPI (Sigma-Aldrich, St. Louis, MO). Slides were subsequently imaged using a confocal microscope with a 10× objective (Zeiss LSM 780; Zeiss, Jena, Germany).

### *In Vivo* Electrophysiological Recordings

Electrophysiological data (from 30 sessions across animals) were acquired at a sampling rate of 10 kHz through wireless headstages with a bandwidth of 1 Hz to 5 kHz (Multi Channel Systems, Reutlingen, Germany). All electrophysiological data were analyzed using custom-written MATLAB scripts (Mathworks, Natick, MA). Raw traces were high-pass filtered (4th order butterworth filter at 300 Hz) and spikes were extracted each time when the trace crossed the threshold of −4-times the standard deviation (2 ms deadtime). Trials exhibiting clear artifacts in the broadband trace were excluded from the analysis.

### Single-Unit Analysis

Spikes were sorted into putative single units (SUs) based on similar waveform characteristics using k-means overclustering and subsequent linkage analysis^30^. First, a subset of spike waveforms (5000 spikes) in a session was sorted into a large number of groups using k-means clustering. Next, similar spike waveform clusters were combined using linkage analysis in order to create templates of single unit waveforms. Finally, all spikes within the session were matched and sorted into the waveform templates. Corresponding spike times were extracted for subsequent analyses. Previous studies have revealed differences in action potential waveform duration, measured by the peak-to-trough time, between regular-spiking (RS) pyramidal and fast-spiking (FS) interneurons^31^,^32^. To determine the cutoff point for the classification of RS and FS units, we calculated the 10^th^ percentile value of the distribution of spike peak-to-trough durations. This threshold was motivated by the non-normal property of the distribution (Kolmogorov-Smirnov test comparing to normal distribution, p < 0.0001) and its heavy tail towards short durations. Accordingly, we classified putative RS and FS units by peak-to-trough durations of greater than or less than 0.52 ms, respectively. The peri-event time histograms (PETH) of the firing rate (FR) were calculated using bin widths of 200 ms for each trial. The trial PETHs were then z-score normalized by subtracting the mean baseline FR (calculated from the preceding seven second portion of the intertrial interval) from each bin and dividing by the standard deviation.

### General Linear Model

We used a multi-variable linear model to explain how task conditions contribute to the variability in single unit firing response and to identify single units that displayed differential firing rates as a function of the trial type^33^. For each single unit, a general linear model was generated for each time bin of the analysis window. The linear model was set up using the following equation:




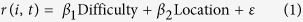




where the dependent variable *r(i, t*), the z-scored firing rate values across trials for single unit *i* at time bin *t*, is modeled as the linear combination of the independent task variables, conditions *Difficulty* and *Location* across trials. The β coefficients were used to create regression coefficient time-series; the model included an error term, ε. Task conditions were encoded with the following values: task difficulty (easy = −1, hard = +1) and target location (left = −1, right = +1). The time-series of the regression coefficients derived from the linear model then represented the dynamics of condition preference. For example, a positive task difficulty coefficient value indicated higher firing rate in the hard condition compared to the easy condition in that particular time bin. This approach also provided the linear model F-statistics that corresponded to the regression coefficient time-series. To determine which single units exhibited significant preference, we set a threshold for the minimum number of contiguous significant bins (F-statistics) (function to find contiguous bins adapted from MATLAB File Exchange, David Fass). Specifically, we performed bootstrap analysis (100 times per single unit) of the linear model with shuffled condition identifiers, and pooled the number of contiguous significant bins found. We obtained the 95th confidence interval cutoff as the threshold value of 1 significant bin. Any single unit exhibiting at least 2 contiguous significant bins was deemed to have preference for a task condition. To validate the linear model and shuffle control, we plotted regression coefficient time-series of SUs that passed the contiguous bins criteria in the shuffled analysis; we found substantially fewer SUs (<10% of un-shuffled analysis) and no observable pattern in the peaks. We included an interaction term to the linear model in a subsequent analysis, and identified which SUs exhibited a significant main effect and interaction.

### Clustering Analysis

We clustered regression coefficient time-series into groups with similar preference dynamics. Our primary approach was to use hierarchical linkage clustering and dendrogram analysis on the coefficient peak times of units grouped by cell type and condition (positive or negative coefficient peak) of preference (Supplementary Fig. S3). We used this method to capture the salient feature that the preference time-series typically peaked at a given moment in time of a trial. In contrast, clustering of the whole time-series de-emphasizes these peaks since it considers the entire trial-duration to which the preference peak is only a small contributor due to its short duration. We first sorted peak times into hierarchical clusters with Ward’s method which minimizes within cluster variance. Using these clusters created from the linkage analysis, we constructed dendrograms to visualize the hierarchical tree. We determined the number of cluster families required to optimally separate coefficient time-series as the number of dendrogram tree leaves at 95% of the full tree. Hierarchical clusters were then merged to create cluster families.

### Support Vector Machine

To test the effectiveness of population decoding of task properties, we performed support vector machine (SVM) analysis on session-averaged population FR data^21^,^22^ for each of the two task variables. FR was calculated using a 400 ms sliding window (100 ms steps) across the analysis window centered at target touch. We utilized a “leave-one-out” algorithm for computing the SVM where, for each FR bin in each session, the model was trained on data from all except one test trial. In other words, the FR data for each bin was a matrix with the dimensions of SU by non-test trials, and was used to predict the trial task conditions (a vector of trial identifiers coding for either difficulty or location). We performed multiple iterations of the SVM such that each trial was tested in the model. Prediction accuracy for each time bin was calculated by taking the ratio of correctly predicted trials to total trials.

We compared population decoding of task variables between session-averaged and session-pooled data. In the former analysis, we performed SVM analysis on each session and took the average accuracy across sessions (average of 20 SUs per session). In the latter analysis, we trained the SVM on pooled SU activity across sessions. In this analysis, we randomly selected 20 trials for each of the two task variables (hard versus easy, left versus right, respectively). The FR data matrix and trial condition vector were organized in such a way that all trials of one condition were positioned in the first half of the matrices and trials of the other condition were positioned in the second half. This method ultimately allowed for assessment of population encoding pooled across sessions in a pseudo-simultaneous manner^34^. Fifteen iterations of the session-pooled SVM analysis was performed in order to include the majority of trials and to perform statistics.

To determine significance, we first calculated chance performance by training the decoder with the same data but with shuffled trial identifiers. After decoding accuracy time-series were calculated, paired *t*-tests were performed for each bin between test data, and shuffle control data. Confidence intervals were calculated across sessions for the session-averaged analysis and across iterations for the session-pooled analysis. Gray significance bars in figures were determined using Bonferroni-corrected paired t-tests.

## Results

### Task Performance

Ferrets were trained to perform a two-choice visual discrimination task adapted from an existing rodent paradigm^26^. To initiate a trial, the animals triggered an IR sensor at the back of the behavioral apparatus. Upon initiation, a pair of images (abstract shapes) was simultaneously presented in the left and right windows of the touchscreen monitor (Fig. 1A). Prior to electrode array implantation, each animal was trained to associate one image (conditioned stimulus: CS+) of an image pair with subsequent reward delivery. Different stimulus pairs were used for different animals. Upon nose-poke touch of the window displaying the CS+, an auditory pure tone (200 Hz, 63.5 dB) was played and a water reward was released at a central lickspout in the back of the behavioral chamber (Fig. 1B). The visual stimuli remained on the screen until animals made a nose-poke to one of the windows. Trials with high and low contrast image pairs were randomly interleaved to assess behavior and dl-FC activity for sensory inputs with high (“easy”) and low (“hard”) signal-to-noise ratios.

We first determined behavioral performance with respect to the task components that varied from trial to trial: task difficulty (easy or hard conditions) and target CS+ location (left or right conditions, i.e. stimulus presentation ipsi- and contralateral to the recording location in left dl-FC, respectively). We predicted, based on the fact that low signal-to-noise sensory stimuli require longer integration times^35^,^36^, that the low contrast condition would result in not only lower accuracy rates but also longer reaction times compared to the high-contrast condition. We indeed found that percent accuracy in the low contrast condition (Fig. 1C, animal 1: 92.3 ± 2.20%, animal 2: 73.5 ± 3.16%, animal 3: 89.4 ± 1.31%; mean ± SEM, n = 12 sessions, n = 24 sessions, n = 11 sessions, respectively) was lower than in the high contrast condition (Fig. 1C, animal 1: 99.5 ± 0.45%, animal 2: 99.0 ± 0.53%, animal 3: 98.4 ± 1.47%; paired *t*-test, p < 0.05, p < 0.001, p < 0.01). Reaction times to touch the CS+ were significantly longer in the low contrast condition (Fig. 1D, pooled across sessions, animal 1: 5.35 ± 0.275 s, animal 2: 5.52 ± 0.203 s, animal 3: 6.22 ± 0.307 s) than in the high contrast condition (Fig. 1D, animal 1: 4.59 ± 0.254 s, animal 2: 3.87 ± 0.120 s, animal 3: 4.82 ± 0.259 s; unpaired t-test, p < 0.05, p < 0.001, p < 0.001). These results support the designation of low and high contrast trials as hard and easy, respectively. We did not find significant differences between reaction time for left and right target locations; however, one animal displayed a significant difference in accuracy with respect to target location (animal 1; left: 92.8%, right: 98.5%; paired *t*-test, p = 0.03). Further, reaction times for left and right windows split by difficulty were also not significant (unpaired *t-*test, left vs. right in easy trials, p > 0.05 for all animals; left vs. right in hard trials, p > 0.05 for all animals).

### Electrophysiological Recordings

**Figure 2:**
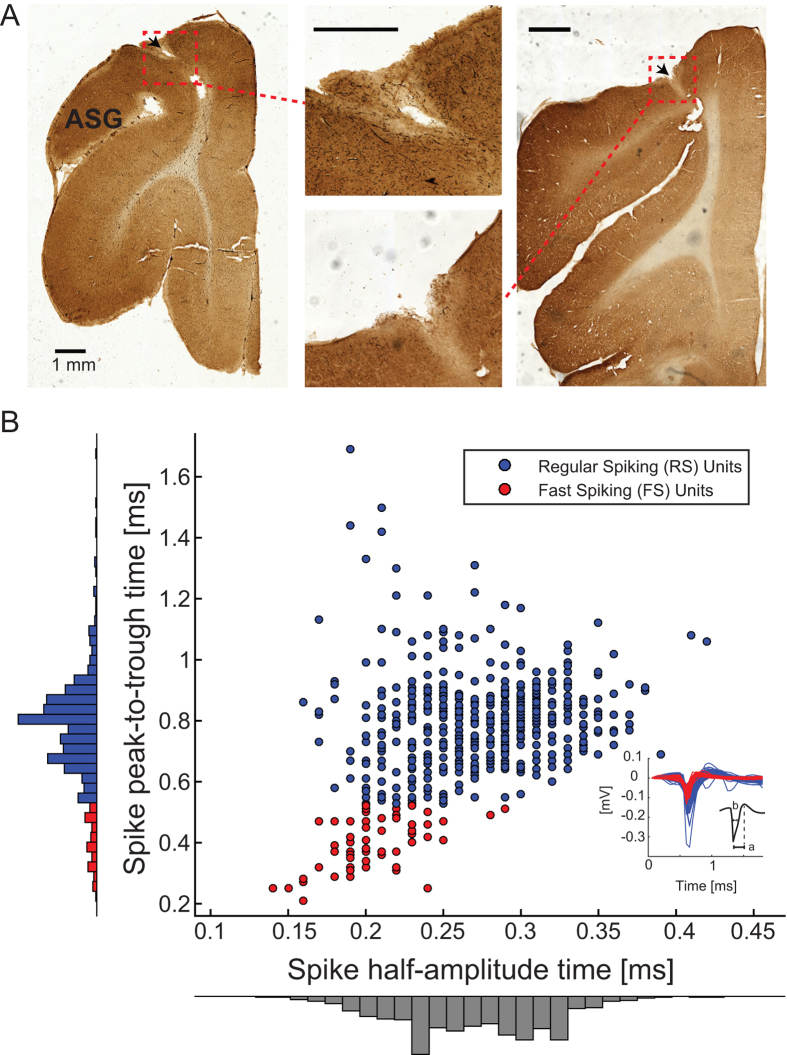
Classification of single unit cell types. (**A**) Coronal sections of FC stained for cytochrome c oxidase for histological verification of recording sites. Left and right images show locations of electrolytic lesions within red boxes. Middle images show electrode insertion site at higher magnification. Black arrows indicate the insertion points of the electrode arrays. Data from two animals are shown, histology from third animal shown in Supplementary Fig. S1. Black scale bars represent 1 mm. (**B**) Action potential duration was characterized by half-amplitude time (x-axis) and peak-to-trough time (y-axis). Single units were classified as presumed regular spiking (RS) or fast spiking (FS) based on the 10^th^ percentile cut-off point in peak-to-trough times. RS units (blue) were identified by long duration (>0.52 ms). FS units (red) were identified by short duration <0.52 ms. Inset: Average waveforms of identified RS (blue) and FS (red) units. Spike peak-to-trough time (a) and half-amplitude time (b) are shown for the black sample waveform.

Electrode arrays were targeted to rostral anterior sigmoid gyrus (1–3 mm from midline) that has been suggested to be the ferret analogue to the primate PFC due to reciprocal connections with mediodorsal thalamus^20^. Electrodes arrays were implanted in the left hemisphere and electrode locations were confirmed through histological methods (Fig. 2A, Supplementary Fig. S1). We refer to this area as dl-FC due to the limited anatomical characterization of the ferret frontal cortex^37^ and continued contention about the definition of PFC across species. We classified single units (SUs) based on action potential duration measured by peak-to-trough time which provides optimal differentiation between regular-spiking (RS) neurons and fast-spiking (FS) interneurons^31^. Putative RS and FS units were identified by peak-to-trough durations of >0.52 ms and <0.52 ms, respectively (Fig. 2B). Out of the total of 587 SUs identified, we found 525 (89.4%) putative RS and 62 (10.6%) putative FS neurons. FS units exhibited significantly higher baseline activity compared to RS units (mean firing rate ± SEM, RS: 2.88 ± 0.004 Hz, FS: 6.56 ± 0.091 Hz, unpaired *t*-test, p < 0.001).

### Task-evoked Modulation of Neuronal Firing Activity in dl-FC

**Figure 3:**
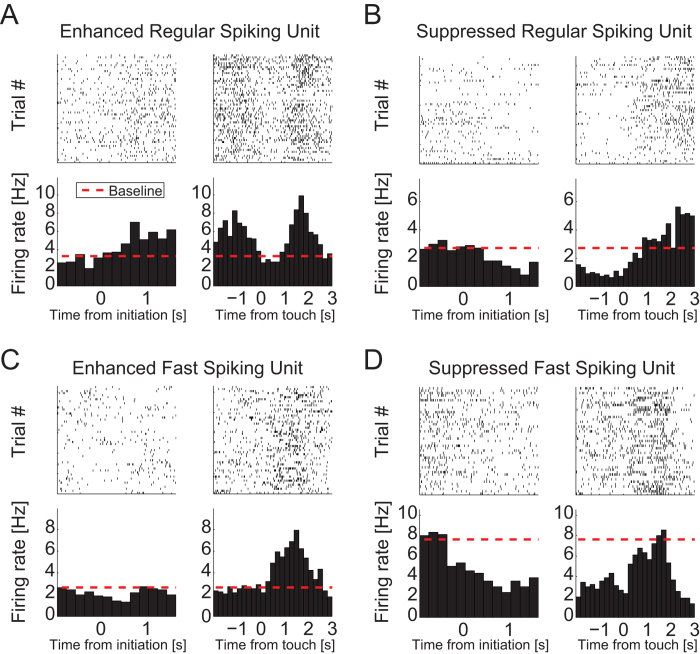
Dynamics of task-modulated single units. Rasterplots and corresponding raw FR PETHs of example task-modulated SUs. Activity is aligned to initiation (left column) and target touch (right column). Plots aligned to initiation and target touch are not continuous due to variable response times across trials. Behavioral epochs are defined, relative to target touch, as follows: stimulus viewing (−2 to −0.5 seconds), stimulus touch (−0.5 to 0.5 seconds), reward acquisition (0.5 to 3 seconds). Dashed red lines indicate average baseline activity levels. (**A**) RS unit with increased activity prior to stimulus touch and during reward acquisition. (**B**) RS unit with activity suppression during stimulus viewing. (**C**) FS unit with increased activity during reward acquisition. (**D**) FS unit with suppression during the stimulus viewing epoch.

Frontal cortex is key to a diverse range of cognitive functions, including stimulus categorization, adaptive decision making, attentional processing, and working memory^1^,^11^,^33^,^38^. We therefore anticipated that neurons in dl-FC would exhibit task-evoked modulation of firing rates (FR) during behavior. As expected, we found modulation of spiking activity in SUs recorded during the behavioral task; averaged across trials, units exhibited both time-locked enhancement and suppression of their firing rate as a function of time. Activity modulation occurred throughout the behavioral task including the pre-touch stimulus viewing and approach (relative to stimulus touch, −2 to −0.5 seconds), touch (−0.5 to 0.5 seconds), and post-touch reward acquisition (0.5 to 3 seconds) epochs (Fig. 3). The task was self-paced; as a result, reaction times from stimulus onset to stimulus touch varied from trial to trial. We performed subsequent analyses exclusively on correct trials. Accordingly, we refer to touching of the correct stimulus as “target touch.”

### Encoding of Task Variables

We first asked if neuronal firing in dl-FC was modulated by task variables difficulty and target location. We defined preference as an elevated firing rate for trials with one versus the other value of a task variable. First, we asked if neurons in dl-FC encode one or both task variables. We performed a general linear model analysis (see details in methods). For each SU, a general linear model was generated for each time bin of the analysis window^33^. We used task conditions as independent variables and FR values across trials as dependent variables in the model. The resulting time-series of regression coefficients represented the dynamics of condition preference; the sign of the regression coefficients indicated which condition was preferred and the magnitude indicated the strength of preference.

We next identified SUs that exhibited contiguous significant time-series bins through bootstrap analysis in which we shuffled trial identifiers. We obtained a 95^th^ confidence interval cutoff value for the number of significant bins required for the SU coefficient time-series to exhibit significant preference for a task condition. Using this criterion, we identified SUs (394 of 587, 67.1%; RS: 66.9%; FS: 69.4%) that had significant preference for at least one of the two task conditions (Table 1). Substantially more cells exclusively encoded location (RS: 165 units, 31.4%. FS: 11 units, 17.7%; 52 of these units exhibited a significant interaction term) than task difficulty (RS: 97 units, 18.5%. FS: 12 units, 19.4%; 34 of these units exhibited a significant interaction term; Chi-square comparing number of units with preference for difficulty or location, Chi-square (1, 285) = 20.8, p < 0.001). We also found units that encoded both task variables (RS: 89, 17.0%. FS: 20, 32.3%; 34 of these units exhibited a significant interaction term). To ensure that these results were not produced by an artifact of the shuffled bootstrap analysis, we plotted the coefficient time-series of spurious “significant” SUs (less than 10% of all units) from the shuffled data and confirmed the absence of a meaningful pattern.

### dl-FC Single Units Encode Task Difficulty

**Figure 4:**
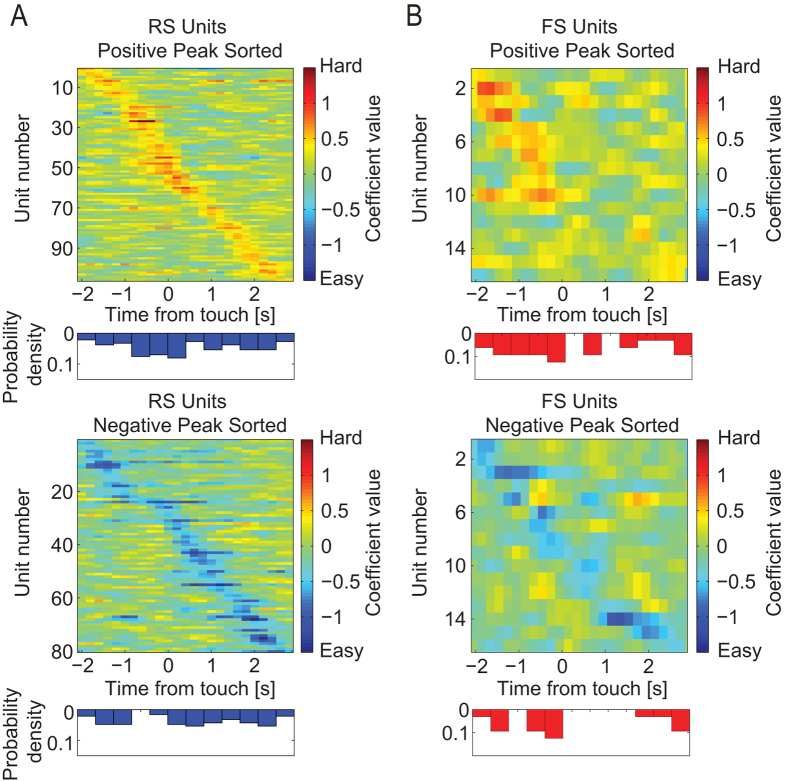
Coefficient time-series of task difficulty preference. (**A**) Heat maps and corresponding probability density functions of regression coefficient time-series extracted from the linear model for populations of RS units with difficulty preference. Positive and negative peak sorted (top and bottom, respectively) time-series heat maps and corresponding probability density functions. Time-series are aligned to target touch. (**B**) FS units exhibiting difficulty preference. Same representation as in Panel (**A**).

First, we studied how dl-FC represents differential task difficulty, i.e. the contrast of the visual stimuli. We hypothesized that the difficult stimuli require more engagement of neurons in dl-FC. To test this hypothesis, we analyzed coefficient time-series of SUs with significant difficulty preference grouped by cell type (Fig. 4, sorted by time of peak value). Indeed, we found a larger number of RS units (Fig. 4A, top, n = 106; Chi-square (1, 186) = 7.3, p < 0.01) that exhibited preferential activity for the hard condition compared to RS units that exhibited preference for the easy condition (Fig. 4A, bottom, n = 80), while the number of FS units that showed preference for either conditions was equal (Fig. 4B, n = 16, for both conditions). This difference indicates a functional dissociation between RS and FS neurons in dl-FC where RS neuron recruitment varied as a function of task difficulty, whereas FS neuron recruitment remained constant.

Then, we asked if the preference for easy or hard trials was maintained through the trial or restricted to a certain epoch. We found that the differential activity peaks of individual units were constrained to certain epochs within trials, and that these epochs were distributed across time at the population level (Fig. 4, blue and red histograms). This suggests the presence of a temporal information-binding mechanism in dl-FC neuron populations that encode task information across the span of the trial. Interestingly, the distribution of peak preference values was not uniformly distributed across time (Kolmogorov-Smirnov test comparing to uniform distribution, p < 0.001). Importantly, units not only showed preferential activity for a given condition prior to target touch (t < 0 seconds), but their patterns of temporally constrained preference peaks also persisted into touch and subsequent reward acquisition epochs (t > 0 seconds). In addition, we observed that a majority of RS units exhibited preferential activity in the hard condition later in time, i.e. shorter time before target touch, in comparison to the RS units that preferred the easy trials (Fig. 4A, top and bottom, unpaired *t*-test of peak times, p < 0.05). Interestingly, about two-thirds of hard-preferring FS units peaked in differential activity prior to target touch; however, the distributions for both FS groups were not statistically different from uniform distributions, likely due to the comparably low number of FS units (Fig. 4B, Kolmogorov-Smirnov test comparing to uniform distribution, p > 0.05 for both). Due to the nature of the task, the time elapsed between stimulus onset (triggered by approaching the lick spout) and stimulus offset (caused by stimulus touch) was determined by the animal. Therefore, the observed sequential patterns could be an artifact caused by different average response times for different sessions such that all units from a given session would correspond to one of the peaks in the preference signal. To exclude this explanation, we plotted the histogram of reaction times for the sessions during which each neuron was recorded from (Supplementary Fig. S2A). These plots show that the behavior was consistent across sessions and that there was no structure that would correspond to the one found for the coefficient time-series, confirming that SU preferential activity indeed reflected the temporal evolution of population activity.

**Figure 5:**
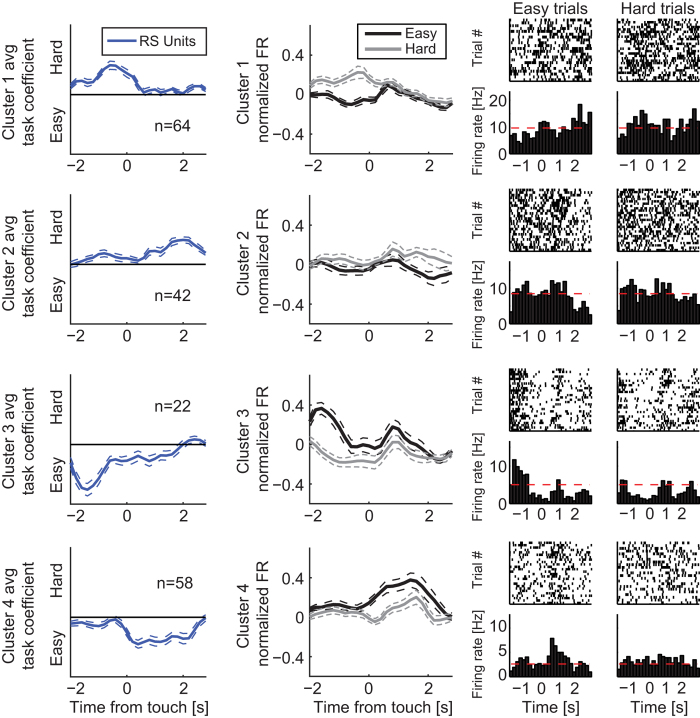
Dynamics of units with difficulty preference. Coefficient time-series, PETHs, and sample rastergrams of RS units clustered by coefficient peak times. Rows correspond to clusters of units grouped by linkage analysis. Left column: Coefficient time-series averaged across units. Middle column: Normalized PETHs averaged across units for easy and hard conditions. Right column: Rastergrams and corresponding PETHs of easy and hard trials for a sample unit in each cluster; dashed red lines indicate average baseline activity levels. All plots are aligned to target touch. Upper and lower dotted lines indicate SEM across units, n indicates number of SUs.

Due to the widespread distribution of preference peaks across time, we further quantified these population preference profiles by clustering SUs according to the time of peak in the coefficient time-series (preference peak time). We restricted our clustering analyses to RS units due to the low FS unit count. We performed linkage clustering analysis to identify SUs that displayed similar preference peak times (Supplementary Fig. S3). We then calculated population-averaged coefficient time-series and FR PETHs for each cluster (separately for easy and hard trials). We found a large cluster of RS units that preferred the hard condition prior to target touch (Fig. 5A, cluster 1: n = 64) and a small cluster of RS units that preferred the easy condition (Fig. 5A, cluster 3: n = 22); cluster 3 exhibited an earlier peak in its preference for the easy condition than cluster 1 in its preference for the hard condition (unpaired *t*-test of peak times, p < 0.01). In the case of cluster 1, preference for the hard condition resulted from an increase and decrease in firing rate for hard and easy trials, respectively (Fig. 5A, mean firing rate in interval [−2 −0.5] seconds relative to target touch ± SEM; hard: 0.15 ± 0.032, easy: −0.04 ± 0.025; paired *t*-test, p < 0.0001, n = 64). The corresponding peaks and troughs in firing rate occurred at the same time (unpaired *t*-test of peak times, p = 0.16, n = 64). In cluster 3, we found the opposite dynamics; preference for the easy condition at target touch resulted from a decrease and increase in firing rate for hard and easy trials, respectively (Fig. 5A, mean firing rate in interval [−2 −0.5] seconds relative to target touch ± SEM; hard: −0.11 ± 0.035, easy: 0.19 ± 0.047; paired *t*-test, p < 0.0001, n = 22). This pattern in differential FR could also be seen earlier in the trial, near trial initiation (Supplementary Fig. S4, cluster 3, mean firing rate in interval [0 2] seconds relative to trial initiation ± SEM; hard: 0.005 ± 0.0290, easy: 0.16 ± 0.037; paired *t*-test, p < 0.001, n = 22). Two further clusters (Fig. 5A, Cluster 2: hard, n = 42; Cluster 4: easy, n = 58) demonstrated significant preference during reward acquisition (paired *t*-test of firing rates in the interval [0.5 2.5] seconds relative to target touch; cluster 2: p < 0.001, cluster 4: p < 0.001). In summary, clusters 1 and 3 contained the neurons that showed pronounced preference for one of the two difficulty levels during the decision-making process and the goal-directed action of touching the stimulus. The number of SUs that were preferentially active during hard trials greatly outnumbered the SUs that preferred easy trials (64 versus 22 SUs).

### dl-FC Single Units Encode Target Location

**Figure 6:**
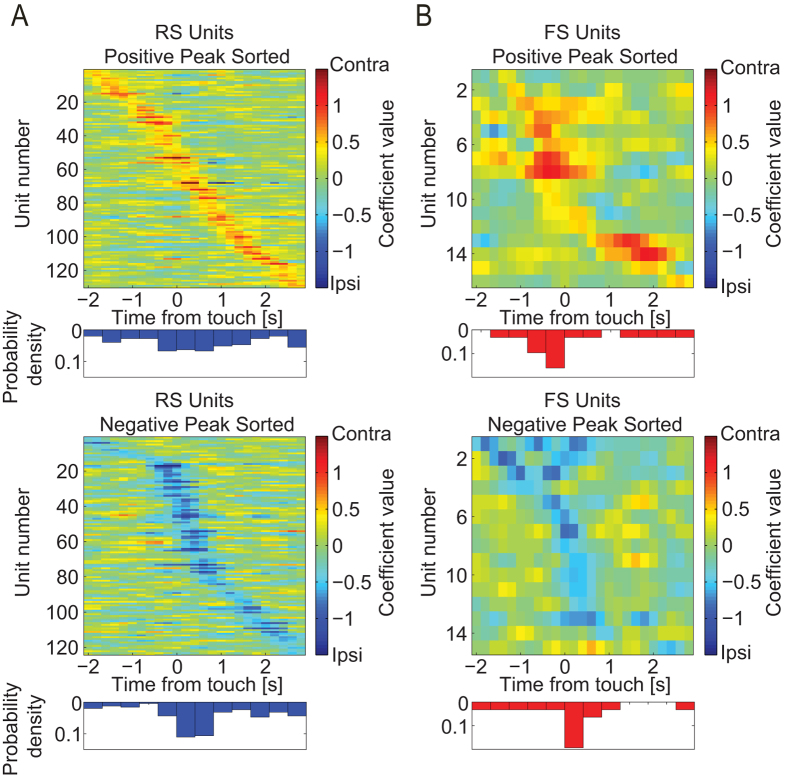
Coefficient time-series of location preference. (**A**) Heat maps and corresponding probability density functions of regression coefficient time-series extracted from the linear model for populations of RS units with CS+ location preference. Positive and negative peak sorted (top and bottom, respectively) time-series heat maps and corresponding probability density functions. Time-series are aligned to target touch. (**B**) FS units exhibiting location preference. Same representation as in Panel (**A**).

Given this bias towards difficult trials, we next asked (1) if the dl-FC neurons that encoded the target location exhibited a similar bias to one of the two locations, and (2) if the target-location encoding exhibited a similar distributed population representation. To answer these questions, we examined the SUs that displayed preference for either of the target locations (Fig. 6). The electrode arrays were implanted in the left hemisphere, thus targets on the left are ipsilateral whereas targets on the right are contralateral. We found similar numbers of ipsilateral and contralateral preferring RS units (ipsilateral: n = 124, contralateral: n = 130; Chi-square (1,254) = 0.28, p = 0.60; Fig. 6A) and FS units (ipsilateral: n = 15, contralateral: n = 16; Fig. 6B) and therefore a lack of differential recruitment of left dl-FC neurons for ipsilateral and contralateral target locations. However, we found distinct dynamics in the temporal evolution of location preference. RS units with preference for presentation of the CS+ in the contralateral window showed peak differential activity in a nearly uniform distribution across the behavioral epoch with modest concentration prior to target touch (Fig. 6A, Kolmogorov-Smirnov test comparing to uniform distribution, p < 0.001). In contrast, nearly half of the SUs with preference for ipsilateral CS+ images exhibited peak preference around the time of target touch (Kolmogorov-Smirnov test comparing to uniform distribution, p < 0.001). Peak time distributions for FS units were not statistically different from uniform distributions (Fig. 6B, Kolmogorov-Smirnov test comparing to uniform distribution, p > 0.05 for both).

We again plotted session-specific reaction time distributions for each SU in the same order as Fig. 6 (Supplementary Fig. S2B), and found no observable pattern. We also examined if extension of the analysis window would alter results: the prescribed results for both difficulty and location heatmaps were unchanged when we performed the same linear model analysis on the window from [−6 3] seconds relative to target touch.

**Figure 7:**
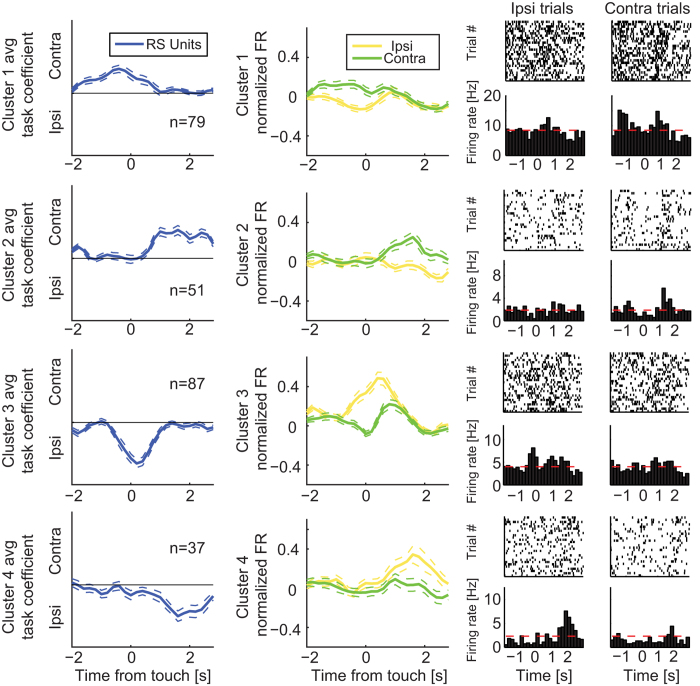
Dynamics of units with target location preference. Coefficient time-series, PETHs, and sample rastergrams of RS units clustered by coefficient peak times. Rows correspond to clusters of units grouped by linkage analysis. Left column: Coefficient time-series averaged across units. Middle column: Normalized PETHs averaged across units for ipsilateral and contralateral conditions. Right column: Rastergrams and corresponding PETHs of ipsilateral and contralateral trials for a sample unit in each cluster; dashed red lines indicate average baseline activity levels. All plots are aligned to target touch. Upper and lower dotted lines indicate SEM across units, n indicates number of neurons.

We then further dissected these population response dynamics for target location preference by clustering the task coefficient time-courses by their peak value (Fig. 7). RS units with contralateral preference (Fig. 7A, cluster 1: n = 79) peaked in location preference prior to target touch, while units with ipsilateral preference (Fig. 7A, cluster 3: n = 87) peaked later at around the time of target touch (unpaired *t*-test of peak times, p = 0.01). The PETHs of contralateral preferring units (Fig. 7A, cluster 1) suggest that firing activity was suppressed around target touch for ipsilateral trials, while firing activity increased for preferred, contralateral trials. Indeed, there was a significant difference in average firing rate between the two trial types (Fig. 7A, mean firing rate in interval [−2 −0.5] seconds relative to target touch ± SEM; ipsilateral: −0.04 ± 0.023; contralateral: 0.10 ± 0.030; paired *t*-test, p < 0.001, n = 79). We observed the same pattern of differential FR earlier in the trial, closer to trial initiation (Supplementary Fig. S5, cluster 1, mean firing rate in interval [0 2] seconds relative to trial initiation ± SEM; ipsilateral: −0.02 ± 0.017, contralateral: 0.04 ± 0.018; paired *t*-test, p < 0.001, n = 79). For cluster 3, there was the opposite response pattern with an increase and decrease for ipsilateral and contralateral trials, respectively (Fig. 7A, mean firing rate in interval [−1 1] seconds relative to target touch ± SEM; ipsilateral: 0.30 ± 0.037, contralateral: 0.06 ± 0.026; paired *t*-test, p < 0.001, n = 87). For both clusters (1 and 3), firing rate peaks and troughs for the contralateral trials occurred before those of the ipsilateral trials (unpaired *t*-test of peak times, cluster 1: p < 0.05, n = 79; cluster 3: p < 0.01, n = 87). We also found that more RS units exhibited preference for contralateral (n = 51) than ipsilateral (n = 37) trials during reward retrieval (Fig. 7A, clusters 2 and 4, Chi-square (1, 88) = 4.5, p < 0.05).

Next, we were interested in the population of neurons that modulated their activity during the task, but lacked preference for a task component. To examine the activity of these units, we calculated FR PETHs for the remaining non-selective RS SUs (Supplementary Fig. S6, left, n = 174) and FS units (Supplementary Fig. S6, right, n = 19). Interestingly, RS and FS units displayed different firing behaviors. For RS units, the largest increases in normalized firing rate occurred shortly after target touch and reward acquisition (unpaired *t-*test between FR of neurons with peaks within [0.5 1.5] and [2, 3] seconds and FR of all other neurons, both p < 0.0001); in fact about a third of all non-selective RS units peaked in their FR in this early phase of reward acquisition ([0.5 1.5] seconds relative to target touch, 30.5% of total RS units). On the other hand, non-selective FS units exhibited the most concentrated and strongest peak FR changes during the stimulus viewing epoch (42.1% of FS units, unpaired *t-*test between FR of neurons with peaks within [−2 −0.5] seconds and FR of all other neurons, p < 0.0001). We found that RS units exhibited significantly higher FRs compared to FS units within the [0.5 3] second epoch (mean ± SEM, RS: 0.169 ± 0.0004, FS: 0.07 ± 0.002, unpaired *t-*test, p < 0.001). On the other hand, FS units exhibited significantly higher FRs within the [−2 −0.5] second epoch (mean ± SEM, RS: 0.13 ± 0.001, FS: 0.61 ± 0.008, unpaired *t-*test, p < 0.001). In summary, non-selective RS units increased their activity leading up to and during reward acquisition, whereas FS units increased their activity during stimulus viewing. This suggests that non-selective RS and FS units play a role in general reward and stimulus processing, respectively.

### Population Decoding of Task Properties

**Figure 8:**
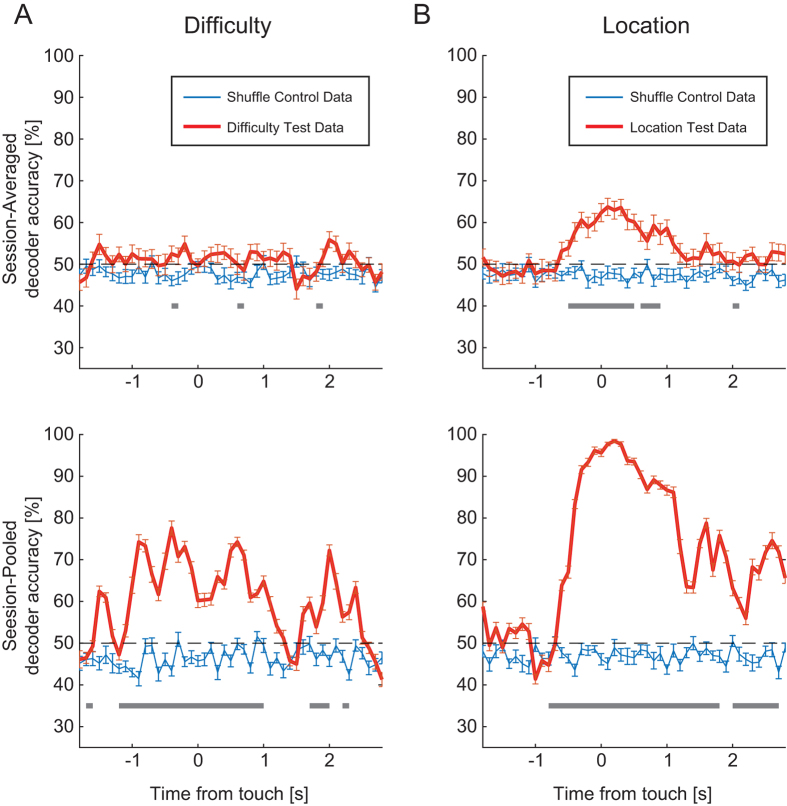
Population Coding of Task Properties. (**A**) Support vector machine analysis was used to establish whether population activity encodes task properties. Top: Session- and animal- averaged decoding accuracies are aligned to target touch. The test data decoding performance is represented by the red line. Shuffle control performance, which was calculated by performing the SVM analysis using shuffled trial identifiers, is plotted in blue. Bottom: Mean decoding accuracy of the session-pooled SVM analysis (SUs pooled across sessions and animals). For each session, a random subset of trials (n = 20) from each condition was included in the model. We computed multiple iterations of the SVM (n = 15) for test data (red lines) and shuffle control data (blue lines). Gray blocks represent statistically significant epochs (paired *t*-test, p < 0.05, Bonferroni-corrected). Error bars, SEM across units. (**B**) Same representations and conventions as in panel (**A**) for location decoding.

So far, we have provided evidence that single units in dl-FC show differential activity for difficulty and location conditions. We next asked if (1) population activity from a given session can be used to decode task variables (difficulty and location) and (2) decoding accuracy improves with population size. To do this, we used support vector machine (SVM) analysis to assess the decoding accuracy of task variables (Fig. 8). In order to address the first question, for each session, we trained the SVM on SU FR data to predict trial conditions (test data). Decoder accuracy was subsequently averaged across sessions (Fig. 8A, n = 30 sessions, average 20 units per session). We compared test data SVM decoding performance to that of a shuffle control (trial identifiers shuffled), and found that population activity poorly encoded difficulty (Fig. 8A top, from [−1 1] centered on target touch, mean = 51%, 95% confidence interval of the mean = 50.6–52.3%, n = 30 sessions), but reasonably encoded location (Fig. 8B top, from [−1 1] centered on target touch, mean = 58%, 95% confidence interval of the mean = 57.2–59.1%, n = 30 sessions).

We next examined decoder performance with population activity pooled across sessions (total of 461 units included). Population decoding performance improved markedly for both difficulty encoding (Fig. 8A bottom, from [−1 1] centered on target touch, mean = 64%, 95% confidence interval of the mean = 63.1–65.9%, n = 15 iterations) and location (Fig. 8B bottom, from [−1 1] centered on target touch, mean = 89%, 95% confidence interval of the mean = 88.1–90.7%, n = 15 iterations). These findings further highlight the distributed nature of the encoding used in dl-FC since most units exhibit preference only for a short window of time and thus a larger number of neurons are required to achieve improved classifier performance. Indeed, the more distributed nature of the encoding of preference for difficulty versus target location that we found with the linear model is reflected in the differential performance of the classifier for the two task variables.

### Inactivation of dl-FC Pyramidal Neurons Affects Visual Discrimination Response

**Figure 9:**
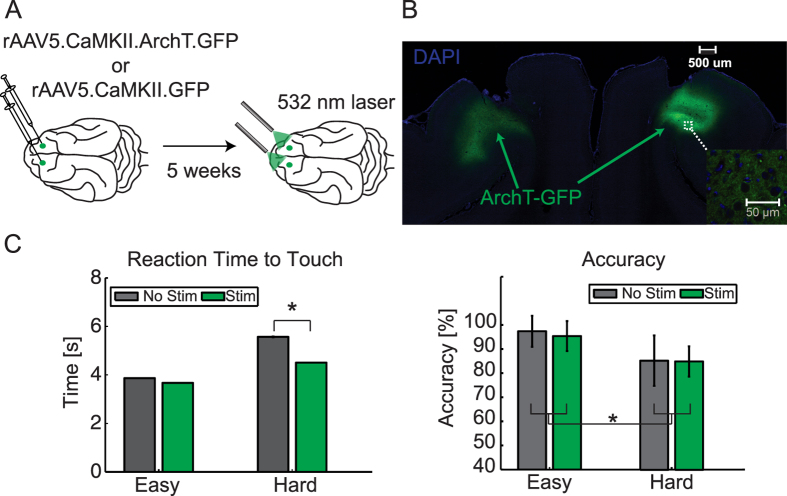
Optogenetics experiment and behavioral performance. (**A**) ArchT experimental design. dl-FC of animals were injected bilaterally with either rAAV5.CaMKII.ArchT.GFP or rAAV5.CaMKII.GFP constructs. Virus was allowed to express for 4 weeks before behavioral experiments. Animals performed the same visual discrimination task as the electrophysiology experiment with the exception that sessions were composed of counterbalanced trials with left/right and stimulation/no-stimulation. Difficulty conditions were designated at the level of session for these optogenetics experiments. During stimulation trials, 532 nm light (15–25 mW) was delivered bilaterally through ceramic-ferrule patch cables during the stimulus presentation epoch (from trial initiation to stimulus touch) or during reward retrieval (from stimulus touch to reward acquisition). (**B**) Coronal section from dl-FC of ArchT animal A for histological verification of virus expression. DAPI stain shown in blue and ArchT.GFP expression shown in green. Inset: 20× close-up of the expression outlined by the white box. Images were acquired with a confocal microscope. (**C**) Accuracy (left) and reaction time to target touch (right) of the ArchT animal in (**B**). Means pooled across trials and sessions for each condition (split by easy/hard and no-stim/stim) shown as bars. Error bars, SEM across trials. *p < 0.05.

To explore the causal role of dl-FC in visual discrimination, we expressed ArchT^24^ (n = 3) or GFP (control animals, n = 2) under the CaMKII promoter and implanted optical fibers in bilateral dl-FC of a different group of animals (Fig. 9A). Expression of ArchT and implant locations were verified in post-mortem tissue histology (Fig. 9B and Supplementary Fig. S7A). These animals performed a similar visual discrimination paradigm with the difference that each session was composed entirely of either easy or hard trials (stimulation/no-stimulation and stimulus location conditions were randomized across trials with each session). We found that when constant light stimulation was delivered to dl-FC during stimulus presentation (from initiation to target touch), reaction time to touch was reduced for ArchT (Fig. 9C, two-way ANOVA, Animal A: main effect of difficulty with F(1, 509) = 56.2, p < 0.0001, main effect of stimulation with F(1, 509) = 13.8, p < 0.001, and significant interaction between stimulation and difficulty with F(1, 509) = 6.58, p < 0.01; Supplementary Fig. S7, Animal B: main effect of stimulation with F(1, 461) = 3.87, p < 0.05) but not GFP control animals (Supplementary Fig. S7B, two-way ANOVA, Animal C: F(1, 546) = 0, p = 0.94; Animal D: F(1, 647) = 1.51, p = 0.22). A third ArchT animal did not show an effect of stimulation on reaction time, but post-mortem histology confirmed that there was only minimal viral expression. One explanation for the reduction in reaction time could be that suppressing dl-FC pyramidal neurons make the animals more impulsive, and as a result impairs decision making. To explore this possibility, we examined performance accuracy across conditions. Surprisingly, stimulation did not alter accuracy (Fig. 9C, two-way ANOVA, ArchT Animal A: F(1, 21) = 0.36, p = 0.55; Supplementary Fig. S7C, ArchT Animal B: F(1, 20) = 0.14, p = 0.71; GFP Animal C: F(1, 20) = 0.06, p = 0.81; GFP Animal D: F(1, 20) = 0, p = 0.96) suggesting that the animals may be over-trained for the assay to show a change in error rates. There was a main effect of difficulty and session when accuracy was plotted as a function of session (Supplementary Fig. 7D, two-way repeated measures ANOVA; for ArchT animals, session: F(1, 5) = 5.2, p < 0.001 and difficulty: F(1, 1) = 27.6, p < 0.0001; for GFP animals, session: F(1, 5) = 5.4, p < 0.001 and difficulty: F(1, 1) = 34.1, p < 0.0001); however, there was no significant effect of stimulation or interaction (Supplementary Fig. 7D, two-way repeated measures ANOVA, p > 0.05 for effects and animal groups). We also found a main effect of stimulation in reaction time when plotted as a function of time for ArchT (Supplementary Fig. 7E, two-way repeated measures ANOVA, F(1, 1) = 4.5, p < 0.05), but not GFP (two-way repeated measures ANOVA, F(1, 1) = 0.67, p = 0.42) animals. Further, we reasoned that the effects on reaction time could either be due to a reduced wait-signal^14^,^15^ or a direct increase in locomotion. To test the hypothesis that stimulation affects pure motor activity, we stimulated ArchT animals during the reward acquisition epoch in “hard” sessions. We found that stimulation during this epoch did not affect reaction time to touch the target (mean reaction time pooled across trials ± SEM; ArchT Animal A, no-stim: 4.94 ± 0.22, stim: 4.64 ± 0.21, unpaired *t*-test, p = 0.38; ArchT Animal B, no-stim: 3.78 ± 0.19, stim: 3.79 ± 0.20, unpaired *t*-test, p = 0.58) or reaction time to drink (mean reaction time pooled across trials ± SEM; ArchT Animal A, no-stim: 1.28 ± 0.003, stim: 1.32 ± 0.003, unpaired *t*-test, p = 0.27; ArchT Animal B, no-stim: 0.96 ± 0.003, stim: 0.94 ± 0.002, unpaired *t*-test, p = 0.94). Thus, if dl-FC pyramidal neuron suppression were to affect locomotion, we would have expected a change in reaction time to drink. These results from the optogenetics experiments suggest a causal role of dl-FC in visual discrimination that is not directly related to locomotion, but rather to the control of task-related responses.

## Discussion

We studied the role of ferret dl-FC in sensory processing and action execution during a visual discrimination task that required freely-moving behavior. In agreement with the well-described role of (P)FC in cognition and behavior, we found pronounced modulation of neuronal activity in dl-FC during the task. We studied how neuronal activity encoded the task variables “difficulty” and “target location” and found that about two-thirds of the units we recorded exhibited preference for one of the two values of at least one of these task variables. Importantly, many more neurons exhibited preference for the hard condition (than for the easy condition) during the pre-touch epoch. With respect to location, we found an equal number of neurons that preferred the left or right target location. However, the temporal patterning of the preference as a function of time was different; a large subdivision of neurons exhibited preference for the ipsilateral condition during the touch epoch whereas preference for the contralateral location was more distributed in time across neurons. Our analysis demonstrated that preferential activation and therefore encoding of task features was dynamic such that most neurons exhibited a relatively short, well-defined time window during which they exhibited such differential firing activity. Across the population, the units exhibited preference at different time-points such that together at any time within the trial, a subset of neurons encoded specific task variables. Indeed SVM analysis further supported this finding of a distributed code. Finally we used optogenetics in freely-moving, behaving ferrets to establish a causal role of neuronal activity in ferret dl-FC during the visual discrimination task. Together, our electrophysiological and optogenetic studies show that hard trials recruit more neuronal activity in dl-FC and that this activity prolonged the reaction time of the animal to touch the target. This suggests that the differential recruitment of neuronal activity in dl-FC for the hard trials does not reflect sensory processing. Rather, the differentially increased activity appears to provide behavioral inhibition that controls other brain structures such that the animal slows down its stereotyped behavioral response, presumably to enable sensory processing of the low signal-to-noise ratio visual input. In further support of this interpretation of our data, we observed a large proportion of active ipsilateral (left) preferring units around target touch. Given the proposed inhibitory role of dl-FC in our study, inhibition of the ipsilateral hemisphere by dl-FC may favor activity in the right hemisphere for successful target touch on the left hand side of the behavioral apparatus.

Our electrophysiology results agree with what is known about PFC function in humans and animal studies, and add an important new dimension given the choice of model species and the fact that our task was designed for freely-moving animals. Our data concur with previous rodent and non-human primate studies that have illustrated the involvement of PFC in sensory decision making^33^,^39^,^40^,^41^,^42^, action selection^33^,^42^,^43^, and motor-related activity^15^,^41^. With regards to dl-FC location selectivity, we observe similar results to previous studies, albeit on a larger time-scale. In non-human primates, neurons that preferentially responded to contralateral targets were activated before those that preferred ipsilateral targets^44^. Similarly, contralateral-preferring neurons came online shortly after stimulus presentation, followed by an increase in number of active ipsilateral preferring neurons^45^. Both studies agree with our findings of target preference in the ferret.

The results revealed in the optogenetics experiments shed further light on the above theories. We found a decrease in reaction time to target touch when dl-FC pyramidal neurons expressing ArchT were stimulated with light. One potential explanation for this decrease in reaction time could be that stimulation increased the speed of locomotion. To address this, we performed ArchT stimulation in the same animals during reward retrieval. We did not find a change in reaction time to retrieve the water reward, suggesting that the stimulation did not simply enhance locomotion. Control animals did not show an effect of stimulation, which ruled out the possibility that the results were driven by the visible light from stimulation or by neuronal heating. An extensive history of studies, mostly in non-human primates, suggests that dlPFC inactivation results in decreased accuracy^8^,^46^,^47^,^48^,^49^,^50^,^51^ and increased reaction time^51^,^52^. There are several possible explanations for the discrepancy in our results. Mechanistically, a large body of literature suggests that dlPFC provides top-down inhibitory control over downstream decision making and motor brain regions^53^,^54^,^55^,^56^,^57^. When dlPFC is inactivated, these inhibitory blocks against goal-directed motor sampling may be lifted^15^, hence the decrease in time to touch the target. A recent transcranial magnetic stimulation study supports this theory by showing that disruption of lateral PFC disinhibited the motor selection of both target and non-target stimuli, and produced a reduction in reaction time^58^. Finally, the optogenetic silencing did not affect behavioral accuracy in our study; this may be explained by how well-trained the animals were on the task.

While several studies have utilized similar touchscreen-based visual discrimination tasks to assay pharmacological manipulations on executive functions^59^,^60^,^61^,^62^,^63^,^64^, little is known about the resulting neural activity patterns. Of particular interest, one study showed that stress or ventro-medial PFC (vmPFC) lesions facilitated visual discrimination during late reversal learning, suggesting that vmPFC provides top-down control of the striatum during performance of the visual discrimination task^64^. Our conclusion based on electrophysiological and optogenetic evidence, that PFC regulates habitual actions through task-relevant inhibition, provides insights into the neural mechanisms of behavioral inhibition.

The opposing locations of the touchscreen and lickspout effectively separated task response from reward retrieval. This represents an important difference to the more classical task design^42^,^65^ in which the animal is head-fixed or provides the response at a feeder where it also retrieves the reward. Our task design allowed us to uncover units that encoded aspects of the stimulus (difficulty, target location) several seconds after completion of the discrimination component and motor response of the task (i.e. after screen touch). The functional meaning of this sustained encoding of these task features remains open to interpretation. Possibly, these late preference peaks after the target response reflect integration of past events for future optimization of behavior to increase reward retrieval opportunities^66^,^67^. Although such sustained encoding is reminiscent of memory cells^1^,^68^,^69^ in PFC, they likely represent a different underlying mechanism. Our task design did not allow for the study of memory cells in the classical sense as there was no retention period with visual stimulation in the task since the two stimuli were presented together on the screen until screen touch. Eye and head position relative to the stimuli in our task may contribute to the underlying neural activity in PFC. However, given that our stimuli spanned a substantial fraction of the visual field since the animals were close to the screen due to the size of the behavioral apparatus, it is unlikely that activation of cells with specific, localized receptive fields play a major role in our findings. Nevertheless, our study is limited by the absence of video-tracking, and we cannot fully rule out the confounding factor of eye and head location. To address this concern with rigor, a new study in the head-fixed animal would be required. We deliberately decided to study the discrimination behavior in the freely-moving animal which actively engages with the stimulus through whole-body movement, an arguably more naturalistic paradigm than the head-fixed preparation. Yet, follow-up studies of similar tasks in head-fixed conditions will provide additional important insights into the neuronal mechanisms of prefrontal cortex activation in visual discrimination.

Anatomical localization of prefrontal cortex in non-primate species remains a question of continued debate. The classical definition uniquely applies to primates and requires the presence of dopaminergic projections, granulation of layer IV, and reciprocal connectivity with the medio-dorsal (MD) nucleus of the thalamus^70^,^71^,^72^. Modified definitions for other species have been proposed although no consensus has been reached^73^. The frontal cortex of ferrets has been only the subject of few studies^20^,^37^,^74^,^75^,^76^. Anatomically, both orbital gyrus and the rostral part of the anterior sigmoid gyrus are connected to the MD nucleus^20^, consistent with connectivity in other species^71^,^77^,^78^. Following this notation, our recordings were localized in dl-PFC on the anterior sigmoid gyrus. However, in keeping with the only other study of ferret frontal cortex during behavior^37^, we designated the recording location as dl-FC due to the poorly understood overall cyto-architectonic organization of ferret frontal cortex and its connectivity.

The only previous study of ferret dl-FC during behavior demonstrated selective responses to visual and auditory signals that were behaviorally relevant^37^. Intriguingly, dual recordings in dl-FC and auditory cortex revealed modulation of inter-area coherence as a function of behavioral context, suggesting that the behaviorally-gated responses in dl-FC contribute to the attention-related, task-optimized top-down modulation of sensory areas. Several key differences to the work presented are recognizable. First, the task used by Fritz and colleagues was a go/no go task in contrast to the alternate forced-choice task as employed in our study. Second, our task was performed by animals that could move freely within the behavioral box (wireless recordings, no head fixation). As a result, our data may be biased towards movement and spatial encoding in contrast to head-fixed experiments. In particular, we found a representation of space that outlasted the actual movement to the target, which provides important insight into the overall functional organization of dl-FC. Despite these differences in task and questions addressed, together these studies motivate the further study of ferret frontal cortex.

### Summary

Our study combined neurophysiological recordings from dl-FC with a visual discrimination task. Despite the extensive use of ferrets as a model study for the developing and adult visual system^79^,^80^,^81^,^82^,^83^, we provide – to our knowledge - the first evidence that ferrets can be easily trained in two-alternative forced choice visual tasks. Of note, we employed abstract shapes as stimuli and therefore were not able to extract basic visual tuning curves such as contrast or orientation sensitivity. Rather, our aim was to understand higher-order cortical processing and how it relates to perceptual difficulty of visual discrimination. We found increased engagement of dl-FC for hard trials where the visual stimuli exhibited low contrast. The use of freely-moving animals combined with the layout of the behavioral chamber enabled us to study neuronal processes that were related to the movement of the animal, in particular the target location. Thus, we provided evidence for neural populations in dl-FC that dynamically process and perpetuate multi-dimensional information throughout the duration of the behavioral task. Finally, through targeted optogenetic silencing of pyramidal neurons during behavior, we demonstrate that activity in ferret dl-FC likely provides top-down inhibition that scales with perceptual difficulty. It remains to be seen if the dynamic encoding of task features generalizes to the more unconstrained real world that lacks the laboratory-controlled trial structure. Nevertheless, our work introduces a new model species for combined electrophysiological/optogenetics and behavioral studies of higher-order cortical function during visual tasks in freely-moving animals.

## Additional Information

**How to cite this article**: Zhou, Z. C. *et al.* Dorso-Lateral Frontal Cortex of the Ferret Encodes Perceptual Difficulty during Visual Discrimination. *Sci. Rep.* **6**, 23568; doi: 10.1038/srep23568 (2016).

## Supplementary Material

Supplementary Information

## Figures and Tables

**Table 1:**
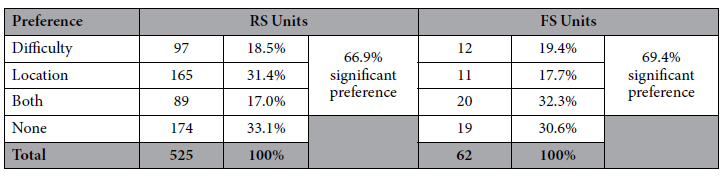
Distribution of single units by cell type and task component preference.

A total of 587 units were included in the analysis. The table summarizes the number of units that were found to exhibit significant (or absence of) preference in the linear model analysis.
