# Supplementary material for: Dorso-Lateral Frontal Cortex of the Ferret Encodes Perceptual Difficulty during Visual Discrimination
Source: Sci Rep. 2016 Mar 30;6:23568. doi: 10.1038/srep23568 (PMC4812342; doi:10.1038/srep23568)
Supplement: Supplementary Information [file srep23568-s1.pdf]

Dorso-Lateral Frontal Cortex of the Ferret Encodes Perceptual Difficulty during Visual Discrimination

Zhe Charles Zhou, Chunxiu Yu, Kristin K. Sellers, and Flavio Fröhlich

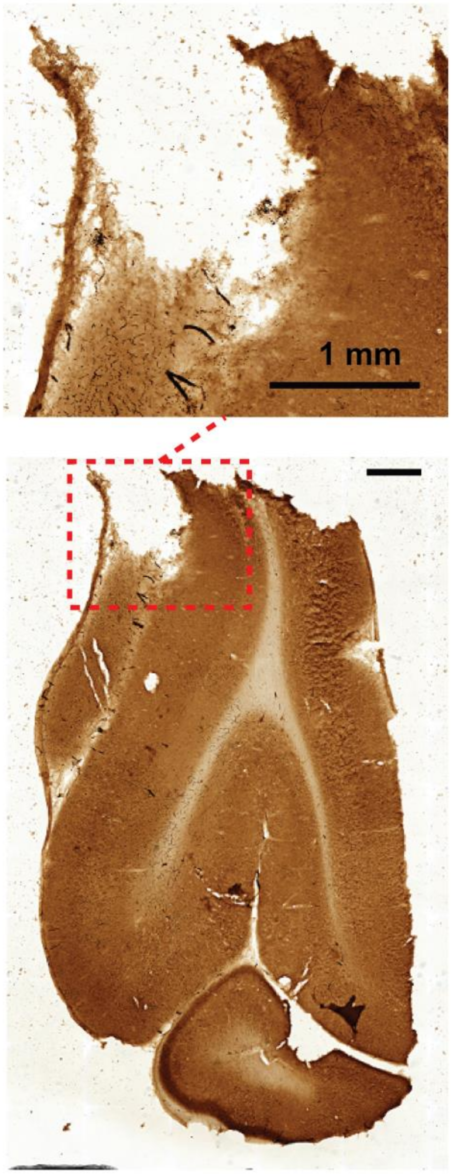

Supplementary Figure S1. Frontal cortex coronal sections of electrophysiology animal 3.

Bottom: Location of electrode array implantation within red boxes. Top: Red box (higher magnification). Tissue damage occurred during electrode array removal after fixation. Black scale bar represents 1 mm distance.

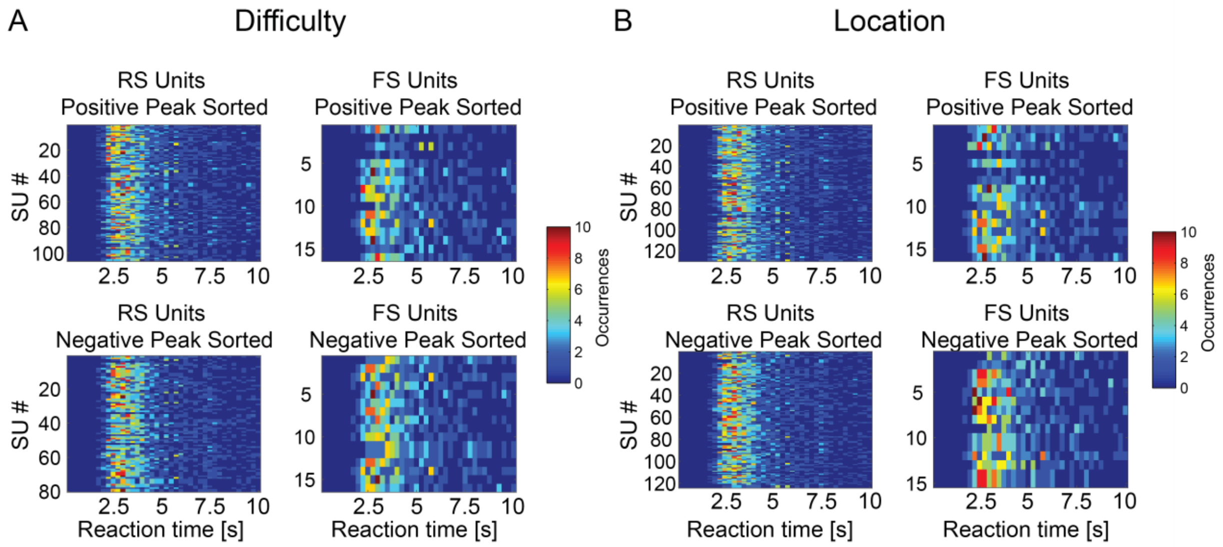

Supplementary Figure S2. Reaction time to touch distributions for sorted units

(A) Session-specific reaction time to touch distributions for difficulty preferring SUs in each group of Fig. 4. Histograms of reaction time to touch were created for each session. To examine the spread of response latencies for experienced by each SU, reaction time histograms were plotted as heatmaps in the same order shown in Fig. 4. Heatmaps were grouped by cell-type and sorting scheme (positive or negative coefficient peak sorted). Colors in the heatmap correspond to the number of occurrences within a bin of reaction time.

(B) Same representation as in (A) for location preferring SUs.

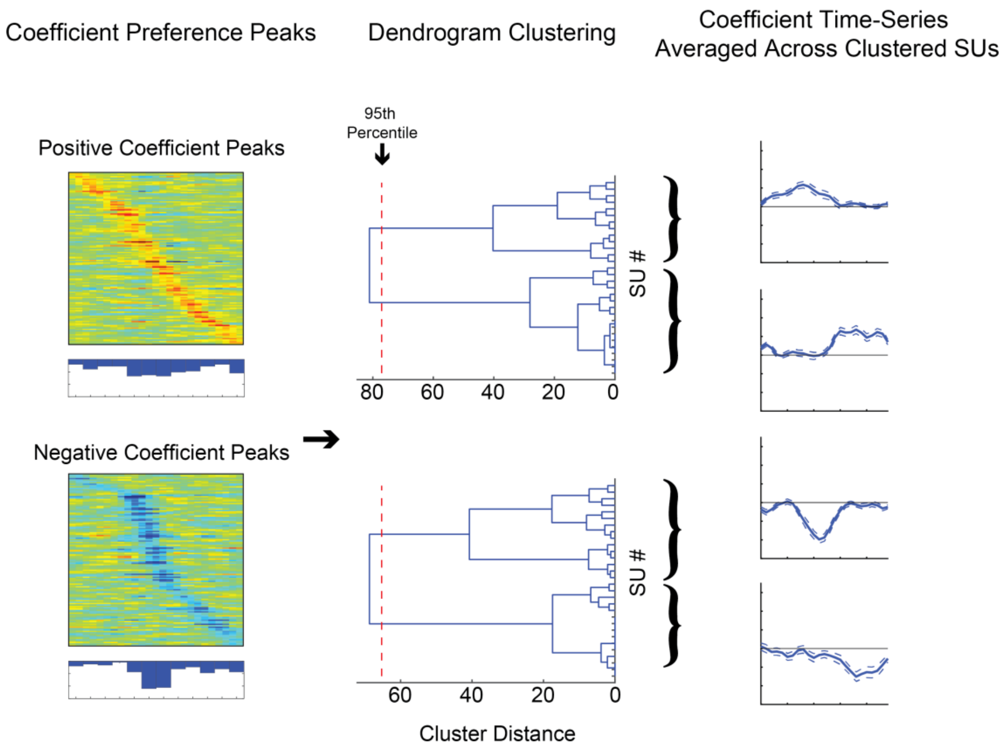

Supplementary Figure S3. Methods for clustering single-units

Due to the diverse, temporally distributed preference peaks, coefficient time-series were clustered by performing hierarchical linkage analysis. Coefficient peak times for SUs in each group shown in figures 4 and 6 (left column here) were hierarchically clustered using Ward's method which minimizes variance in Euclidean distance between clusters. Clusters were visualized by plotting dendrogram trees (middle column) and grouped together where the branches split at the 95th percentile in distance. Coefficient time-series were then averaged across clustered SUs to create plots in figures 5 and 7 (right column).

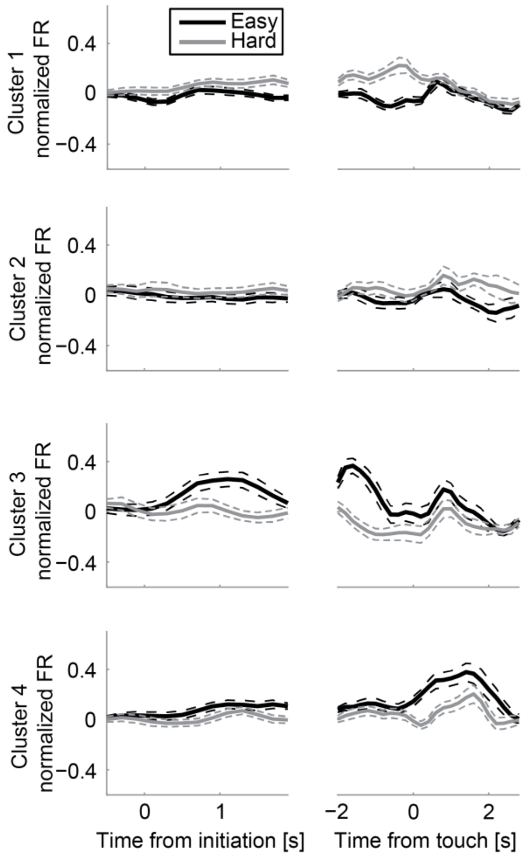

Supplementary Figure S4. Initiation and touch-centered PETHs of difficulty preferring units.

PETHs centered at initiation (left column) and target touch (right column) of units with preference for difficulty clustered by coefficient peak time. Traces are averaged across units for “easy” and “hard” conditions. For all clusters, paired *t*-test of the unsmoothed firing rate traces prior to initiation did not come out to be significant. Upper and lower dotted lines indicate SEM across RS units.

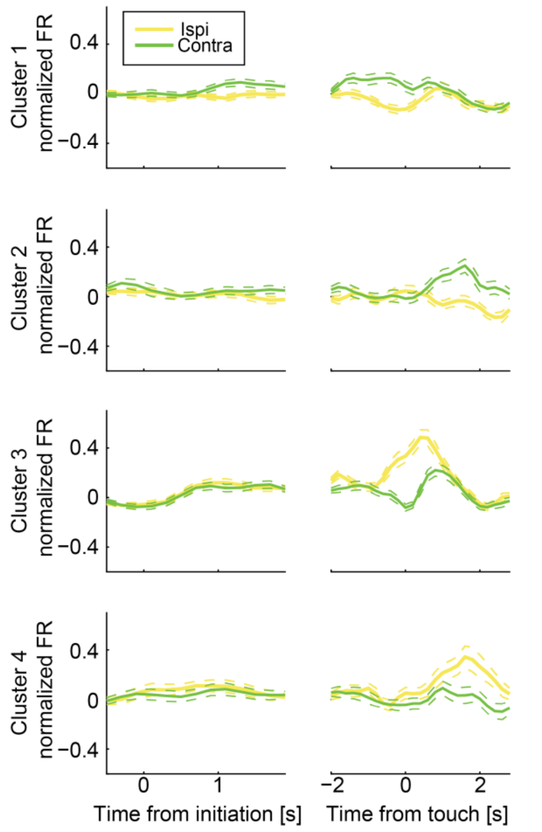

Supplementary Figure S5. Initiation and touch-centered PETHs of location preferring units.

PETHs centered at initiation (left column) and target touch (right column) of units with preference for location clustered by coefficient peak time. Traces are averaged across units for ipsilateral and contralateral conditions. For all clusters, paired *t*-test of the unsmoothed firing rate traces prior to initiation did not come out to be significant. Upper and lower dotted bands indicate SEM across RS units.

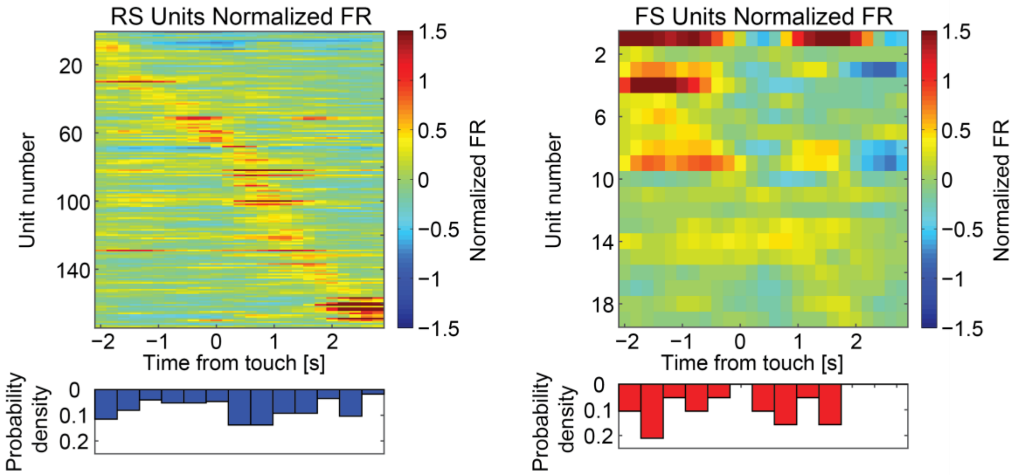

Supplementary Figure S6. Activity of neurons without task variable preference.

(A) Heat maps and corresponding probability density functions of FR PETHs of RS units without task variable preference. PETHs are sorted by peak FR time and aligned to target touch.

(B) Same representation as in (A) for FS units.

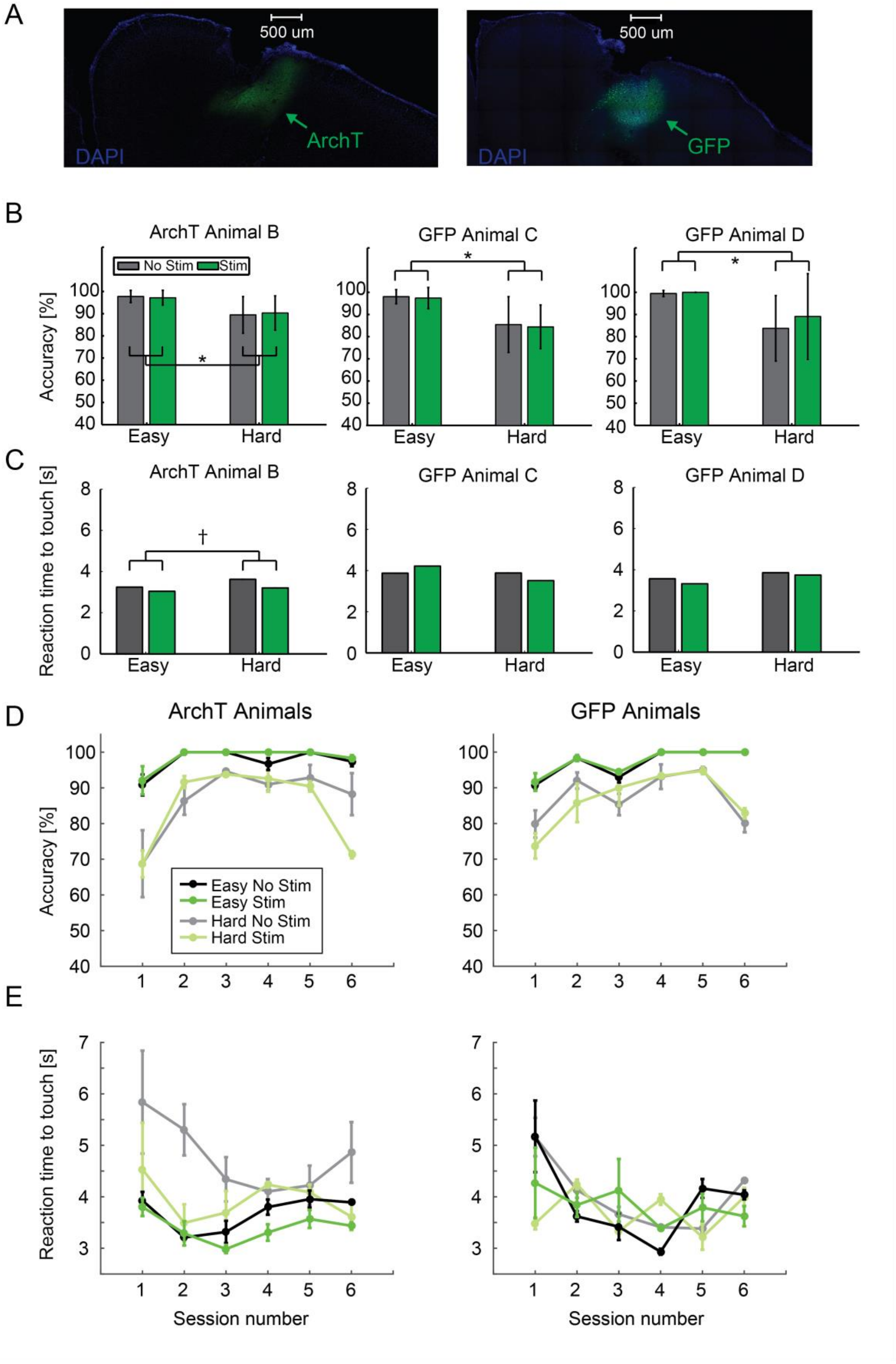

Supplementary Figure S7. Histology and behavioral performance of additional optogenetics animals

(A) Right hemisphere coronal sections from dl-FC of ArchT animal B (left) and a GFP control animal (GFP animal D, right). DAPI is shown in blue, and ArchT or GFP is shown in green. Images were acquired with a confocal microscope with a 10x objective.

(B) Accuracy performance for each condition (split by easy/hard and no-stim/stim) in the optogenetics experiments. Means pooled across sessions for each condition shown as bars. Error bars, SEM across sessions. *$p < 0.05$

(C) Reaction time to target touch for each condition (split by easy/hard and no-stim/stim) in the optogenetics experiments. Means pooled across trials and sessions for each condition (split by easy/hard and no-stim/stim) shown as bars. † For ArchT animal B, we found a significant main effect of stimulation: $F(1,461) = 3.87$, $p < 0.05$. Error bars, SEM across trials.

(D) Accuracy as a function of session. Left: data averaged across ArchT animals (n = 2); Right: data averaged across GFP animal (n = 2). Data is split by difficulty and stimulation conditions. Error bars, SEM across session.

(E) Reaction time to touch as a function of session. Same representation and conventions as D. Error bars, SEM across session.
